# Evidence of large genetic influences on dog ownership in the Swedish Twin Registry has implications for understanding domestication and health associations

**DOI:** 10.1038/s41598-019-44083-9

**Published:** 2019-05-17

**Authors:** Tove Fall, Ralf Kuja-Halkola, Keith Dobney, Carri Westgarth, Patrik K. E. Magnusson

**Affiliations:** 1 Department of Medical Sciences, Molecular Epidemiology and Science for Life Laboratory, Uppsala University, Uppsala, Sweden; 2 Department of Medical Epidemiology and Biostatistics, Karolinska Institutet, Stockholm, Sweden; 3 Department of Archaeology, Classics and Egyptology, University of Liverpool, Liverpool, United Kingdom; 4 Institute of Infection and Global Health, University of Liverpool, Liverpool, United Kingdom; 5 Institute of Veterinary Science, University of Liverpool, Liverpool, United Kingdom

**Keywords:** Behavioural genetics, Human behaviour

## Abstract

Dogs were the first domesticated animal and, according to the archaeological evidence, have had a close relationship with humans for at least 15,000 years. Today, dogs are common pets in our society and have been linked to increased well-being and improved health outcomes in their owners. A dog in the family during childhood is associated with ownership in adult life. The underlying factors behind this association could be related to experiences or to genetic influences. We aimed to investigate the heritability of dog ownership in a large twin sample including all twins in the Swedish Twin Registry born between 1926 and 1996 and alive in 2006. Information about dog ownership was available from 2001 to 2016 from national dog registers. The final data set included 85,542 twins from 50,507 twin pairs with known zygosity, where information on both twins were available in 35,035 pairs. Structural equation modeling was performed to estimate additive genetic effects (the heritability), common/shared environmental, and unique/non-shared environmental effects. We found that additive genetic factors largely contributed to dog ownership, with heritability estimated at 57% for females and 51% for males. An effect of shared environmental factors was only observed in early adulthood. In conclusion, we show a strong genetic contribution to dog ownership in adulthood in a large twin study. We see two main implications of this finding: (1) genetic variation may have contributed to our ability to domesticate dogs and other animals and (2) potential pleiotropic effects of genetic variation affecting dog ownership should be considered in studies examining health impacts of dog ownership.

## Introduction

The relationship between humans and dogs is the longest of all the domestic animals, yet the origin and history of perhaps our most iconic companion animal remains an enigma, and a topic of much ongoing scientific debate^1^. Decades of archaeological and more recent genetic investigations across the world have so far failed to resolve the fundamental questions of where, when and why wolves formed the transformational partnership with humans that finally resulted in the first domestic dog.

Although recent claims for the existence of so-called “Palaeolithic dogs”^2–5^ as early as 30,000 years ago remain controversial^6,7^, there is incontrovertible evidence for the existence of domestic dogs in pre-farming hunter-gatherer societies in Europe at least 15,000 years ago, the Far East 12,500, and the Americas 10,000 years ago^8–10^.

Over the subsequent millennia this ‘special relationship’ developed apace throughout most cultures of the world and is as strong and complex today as it has ever been. Dogs have long been important as an extension to the human ‘toolkit’, assisting with various tasks such as hunting, herding, and protection, as well as for more social activities such as ritual and companionship. The diverse roles that dogs fulfilled most likely introduced a range of selective advantages to those human groups with domesticated dogs. The anthropologist Dr. Pat Shipman went so far as to suggest that the close connection between dogs, other animals and their domesticators had a significant and tangible influence on our bio-cultural history - the *animal connection hypothesis*^11^. A number of experimental studies demonstrate that the view of dogs and other animal stimuli influence human behavior and interest from early childhood onward implicating innate mechanisms^12,13^, whilst others conversely highlight innate adverse responses to spiders and snakes in humans, indicating the evolutionary benefits of avoiding snakes and spiders^14^.

Inspired by assumed physical and psychosocial benefits of dog ownership, pet dogs are now increasingly being used in interventions for the rehabilitation of prisoners^15^, in-patient care^16^ and during pediatric post-surgical care^17^. A large number of studies have shown dog owners to be more physically active^18–20^, leading to acquisition of a dog being recommended as an intervention to improve health. There is also evidence that dog-owners feel less lonely^21^ and have an improved perception of wellbeing, particularly with regard to single people and the elderly^22–24^. We have previously shown that dog ownership is associated with longevity^25^ and lower risk of childhood asthma^26^. However, there are studies showing no relation (or even an inverse one) between dog ownership and these health outcomes^27–29^. One of the important limitations of the available evidence regarding health effects of dog ownership is that it is uncertain whether health differences between dog owners and non-dog owners reflect effects of dog ownership itself, or underlying pre-existing differences in personality, health and genetics. Such factors may impact the choice to acquire a dog in adult life as well as health outcomes – although these factors are difficult to disentangle.

Previous research has indicated that exposure to pets during childhood is positively associated with more positive attitudes towards pets^30^ and ownership in adulthood^31,32^, but it is unclear if genetic differences between families contribute to this association. The heritability of a trait can be estimated from studies comparing concordance of the trait in monozygotic (MZ) and dizygotic twins (DZ) using structural equation modeling. These estimations rely on the underlying assumptions that MZ and DZ twin pairs share environment to a similar degree, that MZ twins share their entire genome, and that DZ twins on average share 50% of their segregating alleles^33^. A previous study of twin pairs aged 51–60 indicated that genetic factors account for up to 37% of the variation in the frequency of pet play and that less than 10% is attributable to the shared childhood environment^34^ indicating a strong contribution of genetic factors to the amount of playful interaction with pets.

Increased understanding of a potential genetic adaption towards dog ownership would support theories of co-evolution of humans and dogs and could also aid the understanding of differences in health outcomes today. However, there are no empirical data supporting a genetic contribution to dog ownership, likely due to lack of information on dog ownership in large twin cohorts. However, it is now possible to study this using register data in Sweden. It is mandatory by law that every dog in Sweden is registered with the Swedish Board of Agriculture. Moreover, all dogs sold with a certified pedigree are also registered with the Swedish Kennel Club. A survey conducted by Statistics Sweden in 2012 estimated that 83% (95% confidence interval (CI), 78–87) of dogs are registered in either or both of the two registers^35^. In this study, we aimed to estimate the heritability of dog ownership in the Swedish Twin Registry, the largest twin cohort in the world.

## Material and Methods

### Study population

The Swedish Twin Registry was founded in the late 1950s and contains information from twins born in Sweden >1886^36^. Twin pairs are systematically identified through population and birth registries and contacted for inclusion in the registry. Zygosity is determined based on intra-pair physical similarities and, for a subset, on DNA testing. In the present study, 93,524 individuals born between 1926 and 1996 were eligible for inclusion.

Information about dog ownership was available from Jan 1^st^ 2001 to Dec 31^st^ 2016 through linkage to the dog registers held by the Swedish Board of Agriculture and the Swedish Kennel Club. We defined the trait dog ownership in a participant as having a dog registered with his/her personal identity number in either of the two dog registers at any time after age 15 during follow-up. This implies that the youngest person in the cohort was aged 20 years at the end of the study period. We excluded 4,042 individuals who had died prior to 2006 and/or those who died before age 20 to ensure a minimum of 5 years follow-up. We further excluded 3,940 individuals with unknown zygosity, resulting in a final analytical data set of 85,542 individuals. These individuals originated from 50,507 twin pairs, where 35,035 pairs included both individuals, and 15,472 pairs included only one individual.

### Statistical methods

We calculated concordance rates and within twin-pair tetrachoric correlations for female and male MZ and DZ pairs, respectively, as well as for opposite-sex DZ pairs. We found dog ownership frequencies to vary with sex and birth-year, and all further analyses were therefore performed with adjustments for these covariates. After initial inspection of data, we decided to model birth year with both linear and quadratic effects.

Structural equation modeling was performed to find maximum likelihood estimates for additive genetic effects A (the sum of the effects of individual genetic variants influencing the trait), shared environmental C (sum of the effects from shared family environment), and unique environmental effects E (sum of the effects from environmental factors specific to each individual). We used the liability-threshold model, wherein the liability of being a dog-owner is assumed to be normally distributed. We found a small difference in tetrachoric correlations across sex when opposite-sex DZ pairs were assumed to have equal correlation as same-sex DZ pairs (p = 0.01). We therefore assessed a series of models with and without qualitative and quantitative sex differences. Qualitative differences means that the genetic factors influencing dog ownership differ for males and females, and quantitative means that the magnitude of effect differs between sexes. The model with lowest Akaike information criterion^37^ without significant deterioration of model fit, when compared to the full ACE sex-difference model, was deemed as the most parsimonious model (further details in Supplementary Methods).

Finally, based on all same-sex twins, we fitted a model where A, C, and E were allowed to vary by age^38^. We defined age as the mean age of each twin during their individual follow-up, and focused on the range 20–75 years old. We adjusted the expected prevalence for age and, because age does not vary between twins in a pair, we did not need to adjust for residual genetic confounding^39^. Thus, the model was based on the classic moderation model^38^, while accounting for phenotype being binary^40^. Because the model had reduced performance towards the extreme ends (i.e., among the youngest and oldest pairs), we used both linear and quadratic moderation parameters. We calculated 95% likelihood intervals with a moving average approach to calculate the bounds.

All twin-based analyses were performed using the OpenMx package^41^ in R^42^.

### Sensitivity analyses

We observed an increase of coverage in the dog register kept by the Swedish Board of Agriculture during the study period. We therefore performed sensitivity analyses by stratifying the follow-up time in two discrete periods, 2001–2007 and 2008–2016. We observed dog-ownership within each period separately, and estimated tetrachoric correlations based on this, while ensuring a minimum of 5 years of follow-up within each time-period.

### Ethical permission

The study was approved by the Regional Ethical Review Board in Stockholm, Sweden (2016/1392-31/1) and participants had given informed consent for inclusion in the Swedish Twin Registry. All research was performed in accordance with relevant guidelines and regulations.

## Results

The participants are described in Table 1. Out of the 85,542 study participants, 8,503 (9.9%) were identified as dog-owners. Dog-owners were more commonly female (65.7%) than in the total study sample (53.7%). The most common dog breed was “Mixed breed” followed by Golden Retrievers and German Shepherds.

**Table 1:** Descriptive information of 85,542 twins born 1926–1996 and alive in 2006 originating from 50,507 twin pairs, where 35,035 pairs included both twins, and 15,472 included only one twin.

	Total (column percent)	Not Dog owner (column percent)	Dog owner (column percent)
Sample (row percent)	85,542 (100.0)	77,039 (90.1)	8,503 (9.9)
Sex			
Male	39,608 (46.3)	36,689 (47.6)	2,919 (34.3)
Female	45,934 (53.7)	40,350 (52.4)	5,584 (65.7)
Birth year			
1926–1939	12,703 (14.9)	12,140 (15.8)	563 (6.6)
1940–1954	23,800 (27.8)	21,388 (27.8)	2,412 (28.4)
1955–1969	16,959 (19.8)	14,303 (18.6)	2,656 (31.2)
1970–1984	13,774 (16.1)	12,053 (15.6)	1,721 (20.2)
1985–1996	18,306 (21.4)	17,155 (22.3)	1,151 (13.5)
County-wide dog density			
<8%	16,413 (19.2)	15,221 (19.8)	1,192 (14.0)
8–10%	29,530 (34.5)	26,901 (34.9)	2,629 (30.9)
10–12%	30,271 (35.4)	26,936 (35.0)	3,335 (39.2)
>12%	9,328 (10.9)	7,981 (10.4)	1,347 (15.8)
Dog breed*			
Mixed breed	2,317 (2.7)	NA	2,317 (27.2)
Golden Retriever	428 (0.5)	NA	428 (5.0)
German Shepherd	389 (0.5)	NA	389 (4.6)
Labrador Retriever	374 (0.4)	NA	374 (4.4)
Dachshund	342 (0.4)	NA	342 (4.0)
Jack Russel Terrier	253 (0.3)	NA	253 (3.0)
Swedish Elkhound	194 (0.2)	NA	194 (2.3)
Cavalier King Charles Spaniel	185 (0.2)	NA	185 (2.2)
Cocker Spaniel	170 (0.2)	NA	170 (2.0)
Border Collie	147 (0.2)	NA	147 (1.7)

Note: All variables are statistically significantly different between dog owners and non-dog owners, at p < 0.001 by Pearson chi-square tests. *Dog owners may have had several different breeds. Only most common breeds reported in this table.

MZ twins had higher concordance rates and tetrachoric correlations (0.58 for females and 0.52 for males) than DZ twins (0.35 for females and 0.30 for males), in line with presence of genetic effects (Table 2).

**Table 2:** Observed concordances and tetrachoric correlations for dog ownership within 35,035 twin pairs.

	No. complete pairs	No. pairs discordant dog owner	No. pairs concordant dog owner	Concordance rate	Tetrachoric correlation
MZ female	6,660	935	306	0.40 (0.36–0.43)	0.58 (0.54–0.62)
MZ male	5,178	501	100	0.29 (0.24–0.33)	0.52 (0.45–0.58)
DZ female	6,801	1,135	193	0.25 (0.22–0.28)	0.35 (0.29–0.40)
DZ male	5,957	691	74	0.18 (0.14–0.21)	0.30 (0.23–0.38)
DZ opposite-sex	10,439	1,731	171	0.16 (0.14–0.19)	0.20 (0.15–0.25)

**Figure 1:**
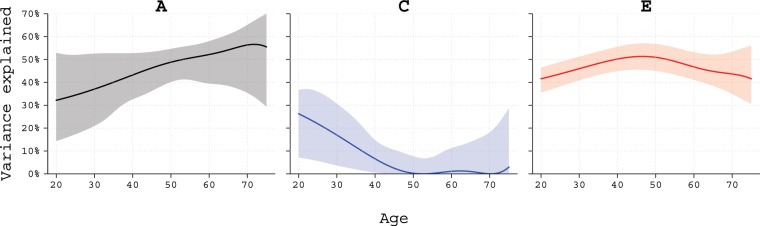
Estimates and 95% likelihood intervals of the contribution of additive genetic effects (**A**), common/shared environmental (**C**), and unique/non-shared environmental effects (**E**) for the trait dog ownership over mean age in twin pairs during follow-up. Note: Due to modelling instability the 95% likelihood intervals are not entirely smooth and a moving average for calculations of the bounds has been employed.

Results from structural equation modelling are presented in Table 3. The estimates for the heritability (A) varied between 48% and 57%, with only minor influence indicated for shared environment (C; range 0% to 6%), and the remaining variance explained by non-shared environment (E; range 43% to 50%). The most parsimonious model was the AE-model with both quantitative and qualitative sex-differences. In this model females had a slightly higher heritability point estimates (57%; 95% confidence interval [CI], 52–61) than males (51%; 95% CI, 44–57). The opposite-sex DZ twin pairs had a correlation that was 0.57 (95% CI, 0.36–0.77) times the expected correlation based on same-sex twins, supporting the contribution of qualitative sex differences. When modelling the contribution of A, C, and E over age, the relative contribution of A increased throughout ages 20 to 75 (Fig. 1). The contribution from shared environment (C) was present at lower ages, but decreased to zero at about age 50.

**Table 3:** Model fitting and estimates of heritability of dog ownership in 35,035 twin pairs adjusted for sex and birth year.

Model	Model comparison measures			Females (95% CI)			Males (95% CI)			Opposite-sex (95% CI)
	ep	AIC	p	A	C	E	A	C	E	r_fm_
ACE, full sex-limitation	11	−118857.39	NA	50% (36–60)	6% (0–18)	44% (40–49)	48% (28–57)	2% (0–19)	50% (44–57)	0.47 (0.00–0.85)
ACE, qualitative sex-limitation	9	−118858.65	0.255	50% (38–58)	4% (0–14)	46% (42–50)	50% (38–58)	4% (0–14)	46% (42–50)	0.44 (0.00–0.74)
ACE, no sex-limitation	8	−118841.70	<0.001	52% (47–56)	0% (0–0)	48% (44–51)	52% (47–56)	0% (0–0)	48% (44–51)	NA
*AE, full sex-limitation*	***9***	−***118860.48***	***0.635***	***57%*** (***52***–***61)***	***NA***	***43%*** (***39***–***48)***	***51%*** (***44***–***57)***	***NA***	***49%*** (***43***–***56)***	***0.57 (0.36***–***0.77)***
AE, qualitative sex-limitation	8	−118859.94	0.327	55% (51–58)	NA	45% (42–49)	55% (51–58)	NA	45% (42–49)	0.55 (0.35–0.76)
AE, no sex-limitation	7	−118843.70	<0.001	52% (49–56)	NA	48% (44–51)	52% (49–56)	NA	48% (44–51)	NA

Note: CI, confidence interval. ACE, model with A, C, and E sources of variance. AE, model with A, and E sources of variance. ep, number of modelled parameters. AIC, Akaikes information criterion. p, p-value from likelihood ratio test against full sex-limitation ACE model. r_fm_, fraction of expected correlation from same-sex twin pairs observed in opposite-sex DZ twin pairs. NA, not applicable. Most parsimonious model indicated by bold and italic font.

### Sensitivity analyses

The tetrachoric correlations were similar when using information available in the different sub-periods 2001–2007 and 2008–2016 (Supplementary Table 1).

## Discussion

The main finding from the present study was that genetic factors greatly contribute to dog ownership in Sweden, with heritability estimated to be 57% for females and 51% for males. Shared environmental factors only contributed in early adulthood. We see two main implications of this finding: (1) genetic variation may have impacted our ability to domesticate dogs and other animals and (2) potential pleiotropic effects of genetic variation affecting dog ownership should be considered in studies examining health impacts of dog ownership.

### Previous studies

Our findings are in line with a previous study in 614 male twin pairs (mean age 55.4) from the US Vietnam Era Twin Study of Aging^34^. In that study, the response to the question “During the past 30 days, how often did you play with pets” was estimated to have heritability of 37% (95% CI, 28–44%) and <10% due to shared environmental factors. Although that estimate is slightly lower than in the present study, the phenotype and setting is also different.

Previous research has indicated that pet keeping during childhood is associated with more positive attitudes towards pets in adulthood^30^ and pet ownership in adulthood^31^. A study of 14,663 children from the UK birth cohort, the Avon Longitudinal Study of Parents and Children (ALSPAC), showed that mothers that had pets during her childhood was a strong predictor of current pet ownership; experience of the father were not investigated^32^. In our study, the estimated contribution of shared environmental factors, was small and only detected in early adult life. Our results thus indicate that previously reported associations of pet keeping in childhood and adulthood are likely to be partly caused by the shared genetic variation between parents and their children.

### Biological mechanisms

Experimental studies suggest general interest toward live animal stimuli during early childhood^13,43^ with more viewing time spent on pets with certain infant-like facial traits^44^. According to the *animal connection hypothesis*, it is likely that unexplored genetic variations can explain differences in human preferences to keeping pet animals such as dogs^11^. The long relationship between dogs and humans has seen both phenotype and genotypes of wolves transformed to the incredible variation found in modern dogs. As is the case for all domesticates, during their long relationship with humans, selection has impacted the nature and trajectory of their development^45^, the retention of juvenile traits^46^ and for some dogs the ability to digest carbohydrates^47^. However, the selection has not necessarily been unidirectional.

The close connection between humans and their domesticates has almost certainly had significant influence on human evolution, genetics, and behavior through reciprocal influences^11^. One important example of such ‘gene-culture co-evolution’ is the human lactase gene (*LCT*) mutation that enables adults in some human populations across the world to consume fresh dairy products without gastric distress. Genetic and archaeological research has revealed that around 7,500 years ago the *LCT* mutation arose in Neolithic cattle herders in central Europe, providing them a significant selective advantage, as this so-called Linear Band-Keramic culture spread westwards across Northern Europe; where the allele is now common^48^.

Although our current study is the first to provide evidence that human genetic factors may perhaps be involved in our choice to keep dogs, our finding does not inform us as to which genes are involved. Like other personality-related traits, we expect polygenic inheritance^49^. Our findings do not provide firm evidence that dog ownership has been selected for in evolution. The gene variation of traits under high selection pressure in the population become fixed and will hence have low heritability estimates. Moreover, genes influencing dog ownership may be highly pleiotropic, and may have been under selection for other reasons. Whilst it is unclear from the literature whether there are personality differences between pet and non-pet owners^50^, there are a number of studies showing evidence of differences in personality between people who own cats and own dogs^51–53^. Personality measures are also associated with multiple health outcomes^54^. For example, dog people score higher on ‘agreeableness’, and ‘conscientiousness’ than cat people^51^, people with higher ‘conscientiousness’ in childhood and early adulthood are reported to live longer^55,56^ and people with higher ‘agreeableness’ are reported to live longer and be at lower risk of cardiovascular disease^57^. Moreover, we have not identified the genes involved and future genome-wide association studies are required in order to better understand this topic. To tease out whether dog ownership has been under evolutionary selection, comparative molecular genetic studies can be undertaken in populations with different historical dependencies on dogs, which could be achieved through a series of carefully targeted ethnographic case studies. If genetic variation linked to dog ownership were identified, then these could potentially be explored further in the archaeological record using ancient DNA techniques.

### Strengths and limitations

The strengths of our study lie in the large sample size, the continuously updated dog ownership data and the large age span studied. Its limitations, however, include potential misclassification of dog owners as non-owners due to two possible reasons. Firstly, not all owners register their ownership; in a survey in 2012, the registration rate was estimated at 83%^35^. If the propensity to report dog-ownership is positively associated with the propensity of the co-twin and/or differentially affecting twin pairs depending on zygosity type, the estimates may be biased towards an over-estimation of the genetic contribution to dog ownership. However, when analyzing periods with worse coverage (prior to 2008) and better coverage, the associations were very similar, supporting the fact that different levels of coverage does not greatly influence the within twin pair associations. Secondly, each dog is registered to only one owner and as we did not have access to data of partners, by default we are misclassifying non-owners where the spouse or household partner is registered as the dog owner. If which spouse is registered as owner is purely random, we would have under-estimated the similarity between twins in pairs, and thus the heritability and/or shared environment contribution. If women in general are more likely to register a dog, this could explain the observed qualitative sex differences. Another limitation to our study is the possibility of non-random mating with respect to dog ownership, so called “assortative mating”. If partner choice is influenced by similarities in preferences for or against dogs or by similarities in factors such as allergies hindering dog ownership, DZ twins will be more similar in dog phenotype than expected from random mating, which could yield a heritability estimate biased downwards^33^.

## Conclusion

In this large twin study including 35,035 twin pairs, we show evidence of a strong genetic contribution to dog ownership in adulthood. In view of the deep history of animal domestication (the first and oldest being the dog) and our long and changing relationship with them, this evidence may be an important first step in unraveling some of the most fundamental and largely unanswered questions regarding animal domestication - i.e. how and why?

## Supplementary information

Supplementary information

## Data Availability

The data that support the findings of this study were made available from the Swedish Twin Registry and are available on reasonable request after ethical permission.
