# Supplementary material for: Evidence of large genetic influences on dog ownership in the Swedish Twin Registry has implications for understanding domestication and health associations
Source: Sci Rep. 2019 May 17;9:7554. doi: 10.1038/s41598-019-44083-9 (PMC6525200; doi:10.1038/s41598-019-44083-9)
Supplement: Supplementary file 1 — Supplementary information [file 41598_2019_44083_MOESM1_ESM.pdf]

Supplementary material

Evidence of large genetic influences on dog ownership in the Swedish Twin Registry has implications for understanding domestication and health associations

Tove Fall, Ralf Kuja-Halkola, Keith Dobney, Carri Westgarth, Patrik K.E. Magnusson

Statistical models

We analyzed the binary variable dog ownership using the so-called liability-threshold model, wherein a normally distributed liability (of being dog-owner) is assumed. Although the liability is not observable, we assume that dog owners have liability values above an estimated threshold while non-dog owners are below. The observed pair-wise distributions of dog-ownership allows inference of the pair-wise correlation between the assumed underlying normally distributed liabilities. These correlations are the basis of the statistical model, and equivalent to tetrachoric correlations.

In addition, since sex-differences were indicated, by considering sexes in twin pairs we estimated sex-differences; quantitative, i.e., estimates allowed to differ between females and males, and qualitative, i.e., the set of genes explaining variance differs between males and females (inferred from opposite-sex DZ having a lower correlation than same-sex twins). Comparing the full ACE sex-difference model with reduced models where quantitative sex-differences were assumed absent, and where all sex-differences were assumed absent, and similarly where C was assumed absent (referred to as AE-model), we tested whether reduced models explained the data equally well as the full ACE sex-difference model.

The model with lowest Akaike's information criterion[1] and without significant deterioration of model fit compared to the full ACE sex-difference model according to likelihood ratio test, was deemed the most parsimonious model.

Supplemental Table 1. Observed concordance/discordance and tetrachoric correlations, for the full follow-up period and the different sub-periods 2001-2007 and 2008-2016. Complete pair analyses.

Zygosity-sex combinations	No. complete pairs	No. pairs discordant dog owner	No. pairs concordant dog owner	Tetrachoric correlation (95% confidence interval)
**MZ female**				
**2001-2016**	6,660	935	306	0.58 (0.54–0.62)
**2001-2007**	6,660	583	144	0.59 (0.53–0.64)
**2008-2016**	6,332	906	297	0.57 (0.53–0.62)
**MZ male**				
**2001-2016**	5,178	501	100	0.52 (0.45–0.58)
**2001-2007**	5,178	323	46	0.50 (0.41–0.58)
**2008-2016**	4,850	465	99	0.53 (0.47–0.60)
**DZ female**				
**2001-2016**	6,801	1,135	193	0.35 (0.29–0.40)
**2001-2007**	6,801	748	81	0.32 (0.25–0.39)
**2008-2016**	6,263	1,085	186	0.34 (0.28–0.39)
**DZ male**				
**2001-2016**	5,957	691	74	0.30 (0.23–0.38)
**2001-2007**	5,957	474	39	0.31 (0.22–0.40)
**2008-2016**	5,422	627	70	0.32 (0.24–0.39)
**DZ opposite-sex**				
**2001-2016**	10,439	1,731	171	0.20 (0.15–0.25)
**2001-2007**	10,439	1,126	71	0.18 (0.12–0.25)
**2008-2016**	9,738	1,640	161	0.20 (0.15–0.25)

References

1 DeLeeuw, J. *Breakthroughs in Statistics: Introduction to Akaike (1973) Information Theory and an Extension of the Maximum Likelihood Principle*. Vol. 1 599-609 (Springer 1992).
